# Thermo-Mechanical Weathering in Malan Loess Under Thermal Shocks

**DOI:** 10.3390/s25103115

**Published:** 2025-05-14

**Authors:** Yangqing Gong, Yanrong Li, Shengdi He

**Affiliations:** Department of Engineering Geology, Faculty of Geological and Surveying Engineering, Taiyuan University of Technology, Taiyuan 030024, China; gongyangqing0102@link.tyut.edu.cn (Y.G.); liyanrong@tyut.edu.cn (Y.L.)

**Keywords:** loess, heat shock, cold shock, weathering, soil erosion

## Abstract

Extreme climatic conditions characterized by drastic temperature fluctuations exacerbate soil erosion through intensified thermo-mechanical weathering processes. Loess-covered regions are particularly vulnerable to such conditions because of the inherent thermo-sensitivity of loess. A comprehensive investigation of mechanisms of thermo-mechanical weathering in loess under extreme temperature regimes holds critical importance for elucidating soil degradation patterns. It is also essential for formulating mitigation strategies in climate-sensitive loess terrains, especially given the increasing frequency of extreme weather events under global warming scenarios. This study employed integrated physical monitoring experiments and numerical modeling. The evolutionary patterns of temperature fields and corresponding thermal stress distributions in loess subjected to both heat shock (rapid heating) and cold shock (rapid cooling) conditions were systematically examined. The key findings are as follows: (1) Soil temperature variations demonstrate phase-lagged responses to ambient thermal variations during both shock scenarios, exhibiting distinct thermal inertia effects. (2) The spatial distribution pattern of thermal stress is predominantly governed by the temperature gradient within the soil matrix. (3) While the magnitude ranges of thermal stress remain comparable between shock types, their directional characteristics fundamentally differ; heat shocks induce surface compressive stresses and internal tensile stresses, whereas cold shocks generate inverse stress patterns. (4) Compared to heat shock, cold shocks trigger obvious surface degradation through tensile stress-induced failure of particle bonds. These mechanically weakened zones establish favorable conditions for subsequent erosion processes in loess landscapes.

## 1. Introduction

Soil erosion is recognized as a major factor in land degradation and soil productivity loss, threatening ecologically and economically sustainable development [1,2,3]. Especially under the global warming climate, extreme weather events are becoming more frequent, such as the phenomena of extreme high temperatures and extreme low temperatures, which cause sudden temperature changes and can easily lead to thermal shock weathering of soil [4,5,6,7,8]. Mechanical effects of thermal shock weathering may lead to a loose structure of topsoil, which, in turn, facilitates soil erosion. Loess-covered areas are widely distributed all over the world, with fragile geologic environments, which are more susceptible to extreme temperature changes [9,10,11,12]. Hence, it is critical to study the thermal shock mechanism of loess under the extreme temperature conditions for the prevention of soil erosion in loess areas [13].

Numerous studies have examined how temperature variations affect rocks. Heat transfers from the ambient environment to the rock surface via convection and then to the interior via conduction, causing temperature fluctuations and redistribution [7,14]. Due to rock heterogeneity and thermal conductivity, temperature responses vary spatially, creating a time lag between surface and interior heating/cooling [15,16,17]. Rapid climatic changes intensify these temperature shifts, particularly at the rock surface [6,8,18]. The resulting thermal gradients induce uneven expansion/contraction, accumulating thermal stress and triggering thermal shock weathering [6,16,17,19]. It is generally recognized that thermal shock consists of two processes: heat shock (rapid heating) and cold shock (rapid cooling), both generating damaging thermal stresses [8,19,20,21,22,23]. While heat shock typically causes rock damage by compressive stresses at extremely high temperatures (hundreds to thousands of °C) [24], cold shock is more likely to induce tensile stresses and subsequent failure under ambient conditions [24,25,26,27]. Thermo-mechanical weathering ultimately leads to three failure modes: granular disintegration, sheet separation, and exfoliation [7,14,20,28,29,30].

Current studies have primarily focused on the effects of thermal shock on rocks [22,26,27,29], while the mechanisms of the effects on loess are still poorly investigated. In particular, the temperature field evolution characteristics and thermodynamic response of loess during thermal shock have not been systematically explained. Moreover, existing studies have predominantly been confined to the one-dimensional heat transfer models in geotechnical bodies [28,29], lacking comprehensive studies on the two-dimensional heat transfer processes. However, such approaches overlook the inherent anisotropy of loess, making it difficult to accurately capture the soil’s response to external temperature fluctuations [9,10]. Therefore, studying the coupling evolution of the two-dimensional temperature field and thermal stress field of loess under the thermal shock processes holds significant theoretical value. It also provides practical guidance for the prevention and control of soil erosion in the Loess Plateau of China (LPC).

Based on the above literature review, we hypothesize that thermal stress induced by thermal shock may cause damage to the topsoil on the Malan loess slopes, which could trigger greater erosion potential. To verify this hypothesis, a series of laboratory experiments were conducted to monitor the internal temperature dynamics of specimens subjected to heat shock and cold shock through a temperature sensor network. By integrating numerical simulations with theoretical analysis, the thermo-mechanical response mechanisms of Malan loess under temperature–stress coupling conditions were systematically characterized. The research findings not only fill a critical knowledge gap in the thermo-mechanical coupling behavior of Malan loess under extreme temperature weathering but also provide a new scientific perspective for the management of soil erosion in the LPC. It further provides more comprehensive technical support for the ecological protection and sustainable development of the LPC region.

## 2. Methods

### 2.1. Sample Preparation

**Figure 1:**
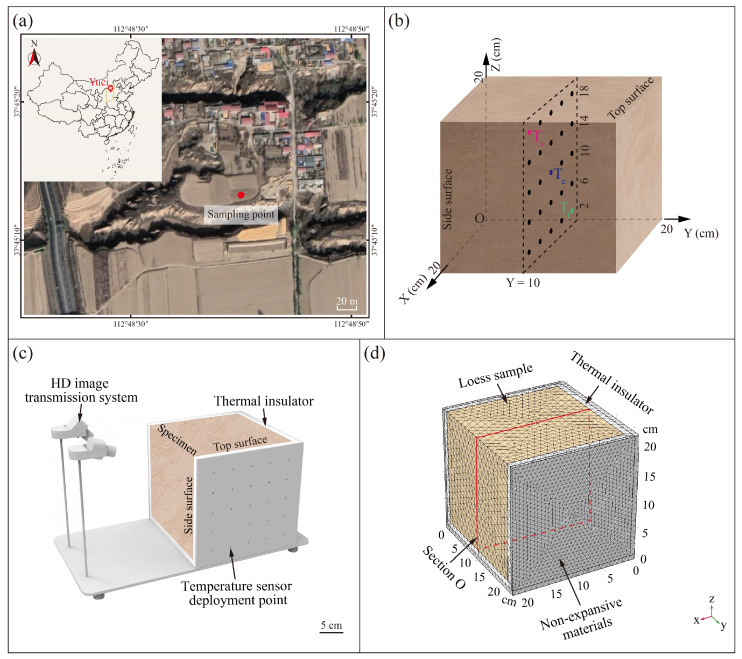
Site location of in situ Malan loess sampling and setup of experiments and numerical simulations for thermal shocks on loess: (**a**) Location of sampling point. (**b**) Specimen size and locations of temperature sensors. *Z*-axis represents the in situ vertical direction, and *X*-axis and *Y*-axis represent two horizontal directions. (**c**) Monitoring of soil temperature and imaging. (**d**) Model meshing of numerical simulation.

The undisturbed samples of Malan loess were collected from Yuci District (37°45′13″ N, 112°48′40″ E), Shanxi Province, on the eastern LPC (Figure 1a). This region is characterized by extensive surface exposure of Quaternary loess deposits, predominantly consisting of the Malan loess (Upper Pleistocene) and partially consisting of the underlying Lishi loess (Middle Pleistocene). This area has a typical temperate continental arid climate with an annual mean temperature of 8.7 °C [31]. Six oriented block samples with a size of 30 × 30 × 30 cm^3^ was taken from a 5 m deep trial pit to avoid the plant roots. The specimens were prepared from the block samples by a wire cutting machine at a slow speed. Six identical cubical (20 × 20 × 20 cm^3^) specimens were prepared for the thermal shock tests (Figure 1b). Three replicate samples were used for the heat shock tests, and the other three were used for the cold shock tests.

The basic physical properties of soil specimens were tested according to the Standard for Geotechnical Testing Method (GB/T 50123-2019) [32]. Soil density was measured using the cutting-ring method. The water content of the soil was obtained via the oven-drying method. The specific gravity of soil particles was obtained by the pycnometer method. The soil particle size distribution was analyzed by using a laser particle size analyzer BT-9300HT JT and was described in terms of clay (<5 μm), silt (5–75 μm), and sand (75 μm–2 mm) percentages according to the Standard for Geotechnical Testing Method (GB/T 50123-2019) [32]. The mechanical properties of loess were from Yuan and Wang (2009) [33]. The loess thermal properties were obtained by laboratory tests. All test specimens were blocky, retaining the intact soil structure. Fifteen sets of specimens were prepared to determine the average specific heat capacity of loess by a thermal constant analyzer Hot Disk TPS 1500. Each set consisted of two specimens, each of which had a diameter and height of 60 mm and 20 mm, respectively. The thermal conductivities of the specimens were measured using a thermal conductivity meter XIATECH TC3000E. Considering the anisotropy characteristics of the undisturbed Malan loess [34,35], the thermodynamic parameters in the three directions were tested separately. A total of 15 sets of specimens were prepared, which were equally divided in the X, Y, and Z directions (Figure 1b). Every set consisted of two specimens (50 mm × 40 mm × 10 mm). A low temperature coefficient of the thermal expansion tester (ZRPY-DW) was used to test the loess thermal expansion coefficients, and 15 cylindrical specimens (diameter = 10 mm and height = 50 mm) were prepared and equally divided along X, Y, and Z orientations for testing. The properties of the intact Malan loess were summarized in Table 1.

### 2.2. Indoor Thermal Shock Tests

This study utilized the GDJS-050 environmental test chamber with a temperature control range of −20 to 150 °C. Meteorological records indicate that the eastern LPC regions have suffered from extreme thermal events, with maximum temperatures surpassing 35 °C. The regions exhibited a mean annual temperature of around 12 °C. For the heat shock tests, the ambient temperature was instantaneously increased from the mean to the maximum, while for the cold shock tests, the reverse setting (maximum to mean) was applied. The rate of increase and decrease in temperature was respectively kept consistent.

In order to understand the effects of ambient temperature on the loess slope surfaces during thermal shock variations, the upper and one of the side surfaces of the cubic specimens were exposed, and the rest of the surfaces were isolated from air contact by a thermal insulator. The configuration of the soil temperature monitoring systems was illustrated in Figure 1c. The test systems comprise a fixed bracket (stainless steel), 25 thermocouple temperature sensors (0.5 mm in diameter with an accuracy of 0.1 °C), and 2 high-definition industrial cameras (1920 × 1080 pixels resolution). Figure 1b presents the specimen orientation and the internal temperature sensors layout within the specimen. These sensors were uniformly mounted on the midplane of the specimen in order to obtain a complete characterization of the temperature field variations in loess in the two-dimensional profile. T_s_, T_c_, and T_i_ were chosen to clarify the characterization of the temperature curves with time from the outside to the inside of loess. The assembled monitoring systems were then transferred into the environmental chamber for the thermal shock tests. The data acquisition systems were programmed to record the ambient and soil temperatures and images at 10-s intervals.

### 2.3. Numerical Simulation

COMSOL Multiphysics 6.0 is a multiphysics field simulation software widely used for numerical simulation and analysis of complex engineering problems. It can effectively deal with the coupling of various types of physical phenomena, including heat transfer, fluid dynamics, solid mechanics, etc. It is particularly suitable for the behavior of geotechnical body materials and their corresponding responses under different environmental conditions [20,36]. Therefore, in this study, the temperature field and thermal stress field of loess blocks were numerically simulated using COMSOL Multiphysics software in order to deeply explore their thermophysical processes and corresponding mechanical behaviors [37]. In this paper, the geometrical model of the indoor thermal variation test was established (Figure 1d), and appropriate physical field modules were selected, including the heat transfer module and the solid mechanics module, in order to accurately simulate the coupled behaviors of heat conduction and thermal stress. The heat transfer module was established based on Fourier’s formula and the energy conservation equation, and the differential control equation for the three-dimensional unsteady thermal conductivity of the anisotropic soil structure under the condition of no internal heat source is given by the following equation [36,37]:

(1) $$\rho c\frac{\partial T}{\partial t}=\frac{\partial }{\partial x}(\lambda_{x}\frac{\partial T}{\partial x})+\frac{\partial }{\partial y}(\lambda_{y}\frac{\partial T}{\partial y})+\frac{\partial }{\partial z}(\lambda_{z}\frac{\partial T}{\partial z})$$

where *T* is the soil temperature; *t* is the time; *x*, *y*, and *z* represent the position of the spatial coordinates; *λ_x_*, *λ_y_*, and *λ_z_* are the thermal conductivities in the *x*, *y*, and *z* directions, respectively; and *ρ* and *c* are the density and specific heat capacity of soil, respectively. All these physical parameters are listed in Table 1.

The transient temperature response inside the soil triggers the thermal stress transient response through the thermal expansion effect, and the thermal stress module is calculated as follows:

(2) $$\sigma_{i}^{T}=E\alpha_{i}∆T_{i}\;\;\;\;(i=x,\;y,\;z)$$

where $\sigma_{i}^{T}$ is the thermal stress tensor; *E* is the modulus of elasticity of loess (Table 1); $∆T_{i}$ is expressed as the difference between the temperatures after thermal change and the initial temperatures in the *x*, *y*, and *z* directions inside the loess; and $\alpha_{i}$ is the thermal expansion coefficients in the *x*, *y*, and *z* directions (Table 1).

In order to improve the accuracy of the calculation, and considering the performance of the computer, we used a finer mesh configuration (Figure 1d) and a transient solver for calculating the temperature field and thermal stress field.

## 3. Results

The heat shock and cold shock tests were respectively conducted using three replicate soil samples. The experimental results showed good consistency among the replicate samples for both testing conditions. For subsequent analysis, one representative set of data from each test was selected to characterize the heat shock and cold shock processes, respectively.

### 3.1. Temperature Characteristics

**Figure 2:**
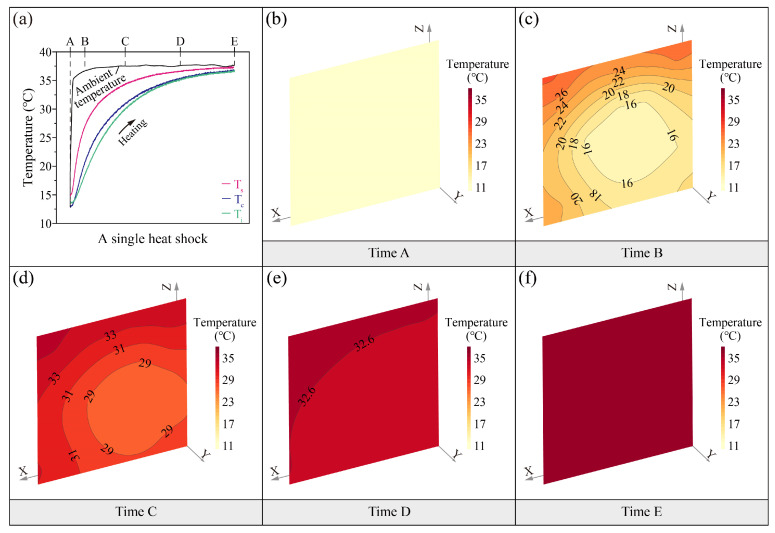
(**a**) Spatiotemporal evolution during a typical heat shock, (**b**–**f**) temperature distributions at five time spots on the section with sensors.

**Figure 3:**
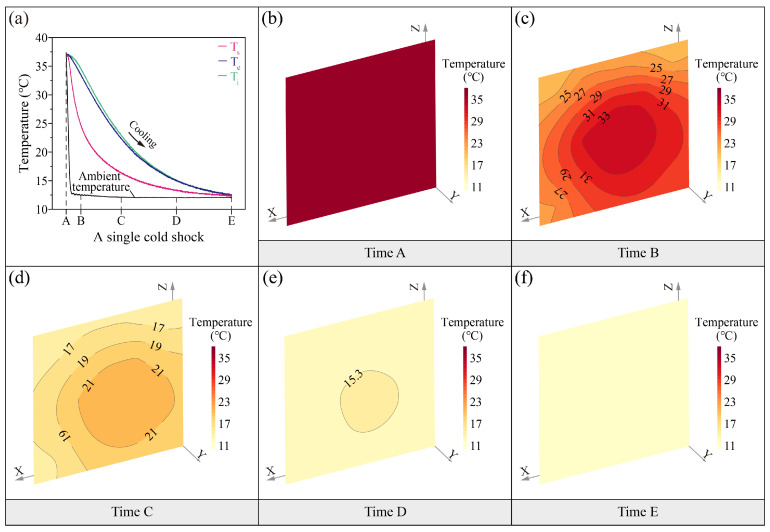
(**a**) Spatiotemporal evolution during a typical cold shock, (**b**–**f**) temperature distributions at five time spots on the section with sensors.

Figure 2a and Figure 3a show the fluctuations of soil temperatures during the different thermal shocks. In the heat shock (Figure 2a), the soil temperatures rise with the ambient temperature. The surface temperature (T_s_) of soil is faster than the center (T_c_) and interior (T_i_). In the cold shock (Figure 3a), soil temperatures decrease with the ambient temperature. The surface soil temperature falls before the center and interior. The soil temperatures under the two thermal shocks exhibit a similar heat transfer pattern. This indicates that loess is extremely sensitive to ambient temperature, with a strong coupling fluctuation relationship between the soil temperatures and the ambient temperature. There is a hysteresis in the temperature transfer from the soil surface to the interior.

The absolute difference in transient temperature between the interior and the surface of the specimen can range from 2 to 12 °C during the heat shock (Figure 2b–f). At the same instant, the temperature of the soil surfaces is higher than that of the interior, indicating that the soil surfaces occur temperature changes before the interior. The isothermal distribution near the soil surfaces is denser than in the interior, suggesting the higher temperature gradients and more drastic temperature changes near the surfaces (Figure 2c,d). Furthermore, the isotherms near the top surface of the specimen are denser than those on the side surface, showing a clear anisotropic characteristic in the heat transfer process (Figure 2c–e). This suggests that the temperature change on the top surface of the soil is more intense compared to the side surface. As the ambient temperature stabilizes, the isothermal distribution gradually changes from dense to sparse, indicating that the internal soil temperature is also gradually stabilizing.

The maximum transient absolute difference in temperature between the interior and the surface of the specimen can reach up to 10 °C during the cold shock (Figure 3b–f). As observed with the heat shock, the surface temperature of the specimen cools down first before the interior. The surface isothermal distributions are intense compared to the interior (Figure 3c,d). The top surface temperature isotherms are denser than the side (Figure 3c,d).

These results confirm that the heat transfer pattern in loess remains consistent during both thermal shock processes, characterized by (1) steeper temperature gradients at the surface compared to the interior and (2) distinct anisotropic thermal distribution.

### 3.2. Thermal Stress Characteristics

**Figure 4:**
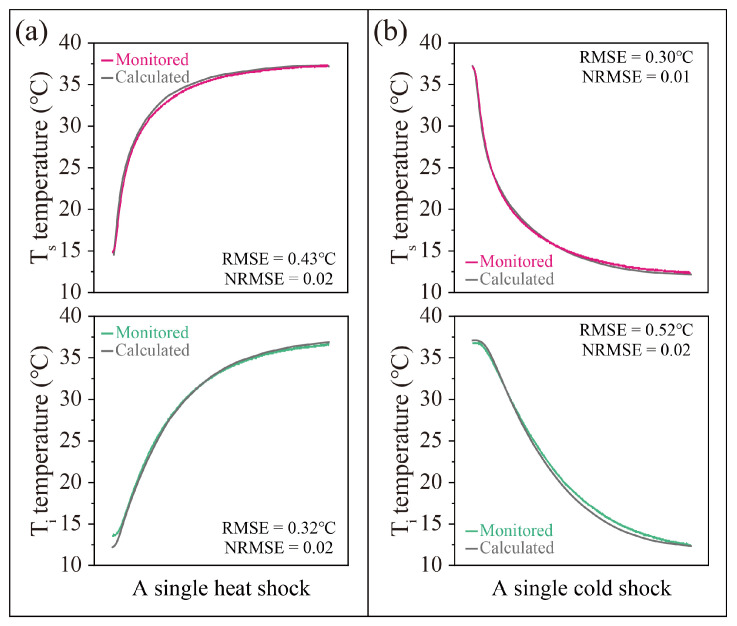
The comparison of soil temperatures from numerical simulation and experimental monitoring during a single heat shock (**a**) and a single cold shock (**b**).

**Figure 5:**
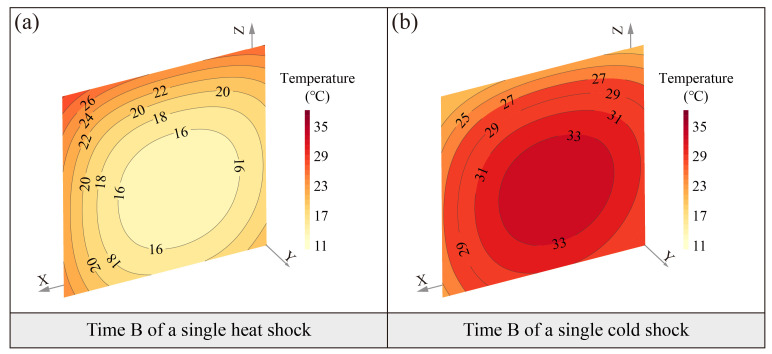
Numerically calculated soil temperature of section O in Figure 1d: (**a**) heat shock and (**b**) cold shock.

Figure 4 shows the comparison of soil temperatures from numerical simulation and experimental monitoring during thermal shocks. The soil surface temperature (T_s_) and interior temperature (T_i_) in the numerical model are consistent with the physical experiments. The deviations between the numerically calculated and experimental monitoring of soil temperature are relatively small, ranging from 0.3 to 0.52 °C for the RMSE and 0.01 to 0.02 for the NRMSE. The temperature field profiles under the different thermal shocks in the numerical results are highly consistent with the physical experiments, which all reflect the drastic temperature changes in the surfaces of the specimen (Figure 5). The above results show that the numerical model has high accuracy in predicting the temperature field; thus, it can provide a reliable basis for the subsequent thermal stress analysis.

**Figure 6:**
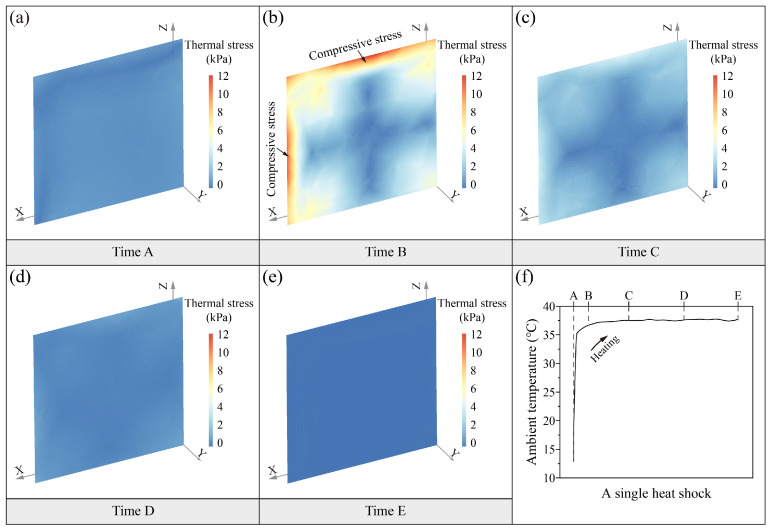
Spatiotemporal evolution (**a**–**e**) of soil thermal stress field in a single heat shock (**f**).

**Figure 7:**
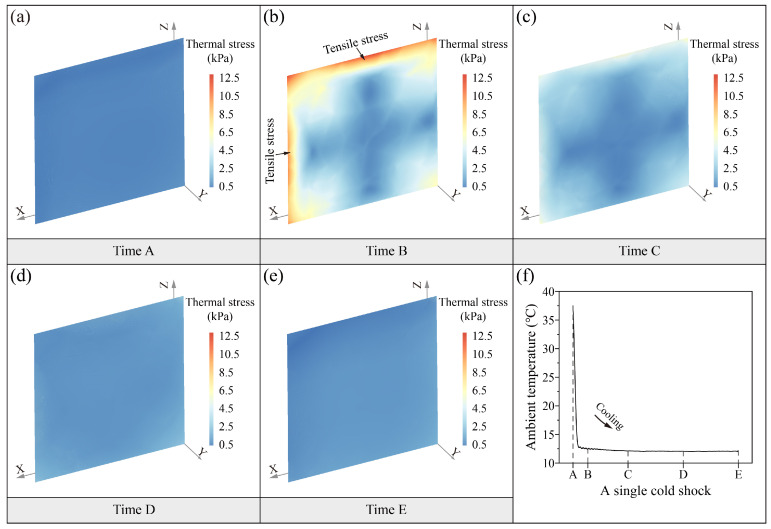
Spatiotemporal evolution (**a**–**e**) of soil thermal stress field in a single cold shock (**f**).

Figure 6 and Figure 7 show the distribution of the soil thermal stress field during the two thermal shocks. The magnitude and distribution of thermal stress during the heat shock and cold shock processes basically show the same characteristics. The range of thermal stress during the two thermal shocks is between 0 and 13.1 kPa. The distribution of soil thermal stresses with time is characterized by instantaneous changes. The soil thermal stress field at the initial moment of thermal shocks (time A) is uniformly distributed (Figure 6a and Figure 7a). After a rapid heating or cooling of the ambient temperature (time B), the thermal stresses in soil surfaces exhibit a concentration of compressive or tensile stresses (Figure 6b and Figure 7b). The surface thermal stresses seem to exhibit subtle differences in distribution. The areas of thermal stress concentration are larger on the top surface than on the side surface. The magnitude of thermal stresses on the top surface is also slightly higher than on the side surface. This suggests that the surface distribution of thermal stresses is characterized by anisotropy. The thermal-induced peak compressive stress is up to 12.2 kPa in the heat shock, while the thermal-induced peak tensile stress is as high as 13.1 kPa in the cold shock. The internal thermal stresses of soil are characterized by a cross-shaped distribution with a lower stress value ranging from 0 to 2 kPa. It is worth noting that the direction of soil thermal stress in the two thermal shocks is different due to the thermal expansion and thermal contraction caused by the heating and cooling. The heat shock process is characterized by the thermal expansion of the soil surface and the thermal contraction of the interior, which results in compressive stress on the surface and tension in the interior. On the other hand, the cold shock process is characterized by the thermal contraction of the soil surface and the thermal expansion of the interior, which results in tensile stresses on the surface and compressive stresses in the interior. At time C, the range of internal thermal stresses in the soil is reduced by a large amount compared to time B (Figure 6c and Figure 7c). The surfaces’ thermal stress is still the highest, and there is a slight thermal stress concentration. The internal cross-shaped distribution of thermal stress characteristics remains constant. As the ambient temperature continues to stay stable, the thermal stresses in the soil gradually decrease with uniform distribution in the last two moments, which range from 0.25 to 2.2 kPa at time D (Figure 6d and Figure 7d) and 0 to 1.6 kPa at time E (Figure 6e and Figure 7e), respectively.

**Figure 8:**
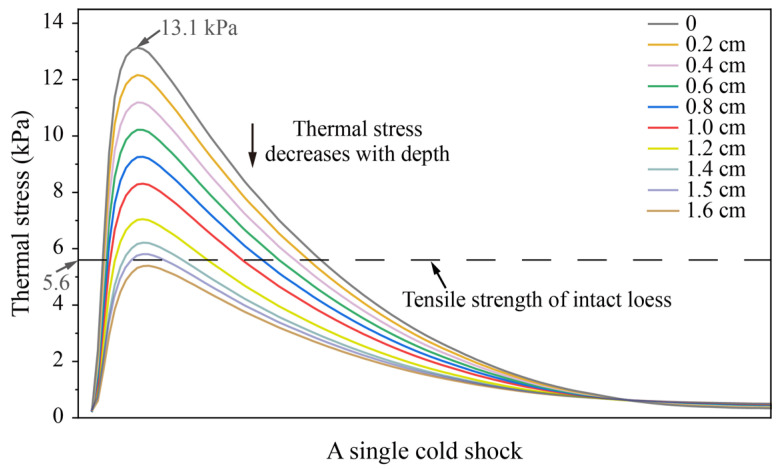
Thermal stresses vary with time along depth of Z direction during a single cold shock.

The distribution of the thermal stress field reveals that thermal stress concentration is present on the soil surfaces during the two thermal shocks. Although the compressive stress generated by the heat shock (~12.2 kPa) is comparable to the tensile stress induced by the cold shock (~13.1 kPa), the latter poses a greater risk of damage because the tensile strength of loess (5.6 kPa for the intact sample used in this study) is approximately one-tenth of its compressive strength [34]. To further analyze the process of thermal stress of the soil surface during the cold shock, the thermal stress versus time curves were drawn, as shown in Figure 8. In a single cold shock, the thermal stress at the soil surface reaches a maximum of 13.1 kPa. The tensile stresses within 1.5 cm of the top surface soil all exceed the tensile strength of the intact dry Malan loess [38]. During the cold shock, the thermal stress in the topsoil is the first to exceed the tensile strength, making it the most likely location for initial damage. The thermal stresses at the soil surface decrease with depth.

**Figure 9:**
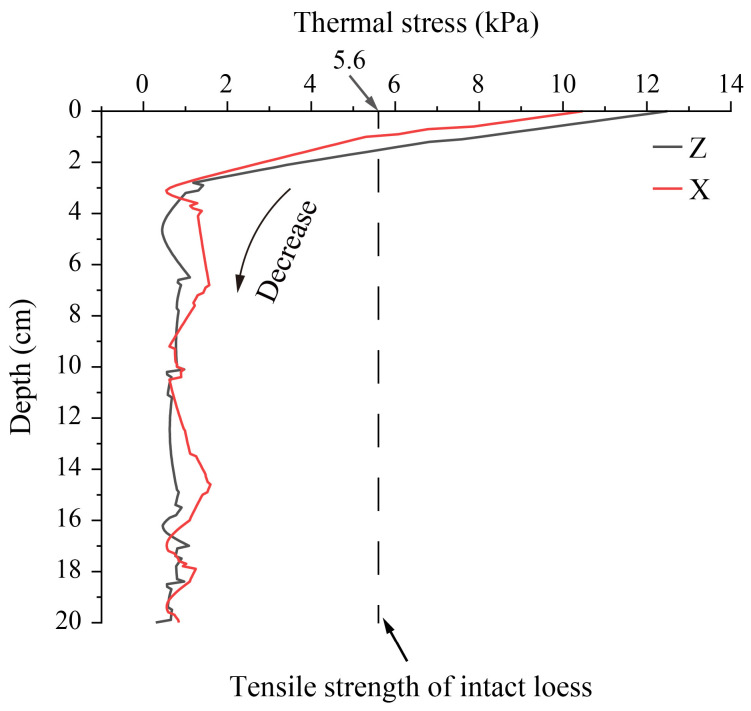
Thermal stress with depth at time B during a single cold shock.

In order to investigate the distribution of tensile stresses over the depth of soil due to cold shock, the curves of thermal stresses with depth at the corresponding moments were drawn (Figure 9). The thermal stress maintains the same pattern of development with depth, both in the Z (vertical) and X (horizontal) directions. The thermal stress first decreases steeply with depth and then remains almost steady. However, there are slight differences in the magnitude of thermal stresses in different directions. At the initial depth, the thermal stress in the vertical direction is significantly higher than in the horizontal direction. After 3.5 cm, the thermal stresses in both vertical and horizontal directions fluctuate around 0, yet the horizontal thermal stress is slightly higher than the vertical direction. Owing to the instantaneous cooling in the cold shock causes the surface soil to contract, resulting in the development of tensile stresses. The internal soil is still in relative expansion due to thermal inertia, resulting in compressive stresses. The higher tensile stress in the vertical direction than in the horizontal direction at the soil surface causes lower compressive stress in the vertical direction than in the horizontal direction within the soil. This indicates the anisotropic distribution characteristics of thermal stress during cold shock. It is further observed that the thermal stresses within 1.5 cm in the vertical direction and 1 cm in the horizontal direction of the soil surface exceed the loess tensile strength. This indicates that the damage depth of cold shock may be 1.5 cm in the vertical direction, while it may be about 1 cm in the horizontal direction, showing obvious anisotropic damage characteristics.

## 4. Discussion

### 4.1. Temperature Gradients of Thermal Shocks

The results of physical experiments and numerical simulations reveal the thermal response characteristics of loess subjected to the two thermal shocks. Soil temperature fluctuates with ambient temperature, which is a common feature of heat transfer in solids [39,40]. The thermal convection between the air and the soil surfaces is the boundary condition for temperature fluctuations in the surface layer. The subsequent temperature gradient is the driving condition for the thermal conduction within the soil. The low thermal diffusivity of loess induces progressive thermal wave attenuation, creating a hysteresis between surface and interior temperature extrema [41,42]. Thus, the heat transfer patterns of heat shock and cold shock in loess are consistent, which are primarily controlled by the thermal convection in the surface soil and the thermal conduction in the interior soil [43].

The difference in temperature fluctuations between the surface and interior of the soil determines the temperature gradient. The temperature gradient not only drives the heat transfer process but also directly contributes to the distribution of thermal stress within the soil. For a general geotechnical structure exposed to the ambient temperature, the internal temperature gradient through the differences in temperature at different locations results in uneven thermal expansion and contraction deformations, and thereby generates the thermal stresses [16,20,24]. Thus, the magnitude and distribution of thermal stresses are mostly influenced by the distribution of the temperature gradient of the soil. The identical thermal stress distribution characteristics during this heat shock and cold shock indicate a large degree of similarity in the temperature gradient of both. This is confirmed by the results obtained from numerical simulations. Hence, the following section highlights the relationship between temperature gradient and thermal stress in cold shock effects and analyzes the effect of temperature gradient on the thermo-mechanical properties of loess.

**Figure 10:**
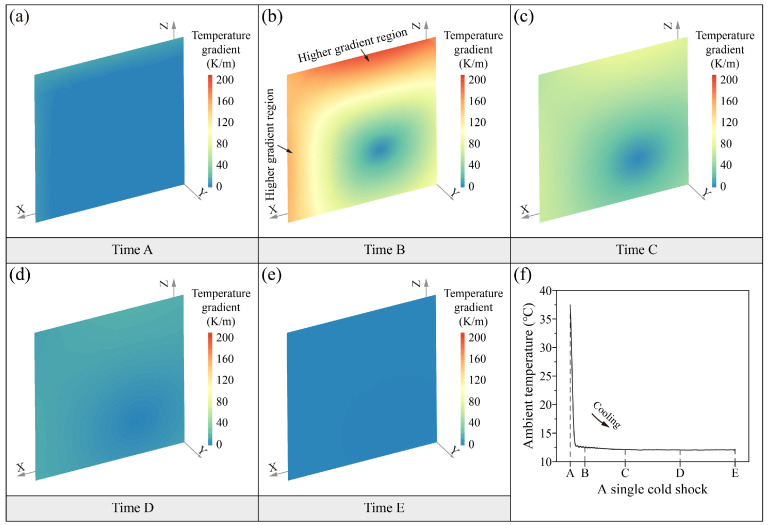
Spatiotemporal evolution (**a**–**e**) of soil temperature gradient in a single cold shock (**f**).

Figure 10 illustrates the spatial distribution of temperature gradients during the cold shock. The magnitude and concentration of thermal stress within the soil are primarily governed by the temperature gradient, as demonstrated in previous studies [44,45]. The numerical simulation results of this study further confirm a strong positive correlation between the two (R^2^ > 0.95). When the temperature gradients within the soil are low (Figure 10a,d,e), the corresponding thermal stresses are also low (Figure 7a,d,e). Conversely, regions with high temperature gradients (Figure 10b,c) exhibit high thermal stresses (Figure 7b,c). Moreover, during the thermal shock process, the temperature gradient distribution within the soil exhibits pronounced anisotropy. Specifically, the average temperature gradient in the vertical (Z) direction (188.5 K/m) is significantly greater than that in the horizontal (X) direction (149 K/m) (Figure 10b), which contributes to the anisotropic distribution of thermal stress within the loess specimen (Figure 7b).

The temperature gradient is usually higher at the soil surface than in the interior under the action of ambient temperature. The high temperature difference between the exterior and interior tends to form a higher thermal stress in the soil surfaces. If the thermal stress approaches the soil strength, it may induce structural damage in the soil [24,46]. This observation not only deepens the understanding of soil thermodynamic behavior but also provides a theoretical basis for predicting and mitigating the damage of surface soil induced by thermal disturbances. For example, in practical engineering, the temperature gradient distribution can be monitored to assess the area of thermal stress concentration so that suitable protective measures can be taken.

### 4.2. Mechanisms of Thermal Shocks

Regarding the significant anisotropy of the thermal stress distribution during the thermal shock, this is mainly controlled by the differences in the thermal conductivity and thermal expansion coefficient in the vertical and horizontal directions of the soil (Table 1). The anisotropy of the temperature gradient is dominantly caused by the vertical and horizontal differences in thermal conductivity. The fundamental reason for the directional differences in the thermophysical properties of loess depends on the intrinsic anisotropic structure. Loess exhibits a characteristic vertiloess structure composed of loess aggregates, fragments, and lumps, which are bound tightly along the vertical direction by strong force chains, whereas the weak segments comprise vertically oriented pipes and cracks that divide the strong units, resulting in a comparatively loose alignment of particles in the horizontal direction [35]. This compositional pattern results in a more compact and continuous structure in the vertical direction, which yields higher thermal conductivity and a higher thermal expansion coefficient compared to the horizontal direction. As a result, the vertical temperature gradient and the vertical thermal stress are higher than the horizontal under the effect of instantaneous thermal changes.

**Figure 11:**
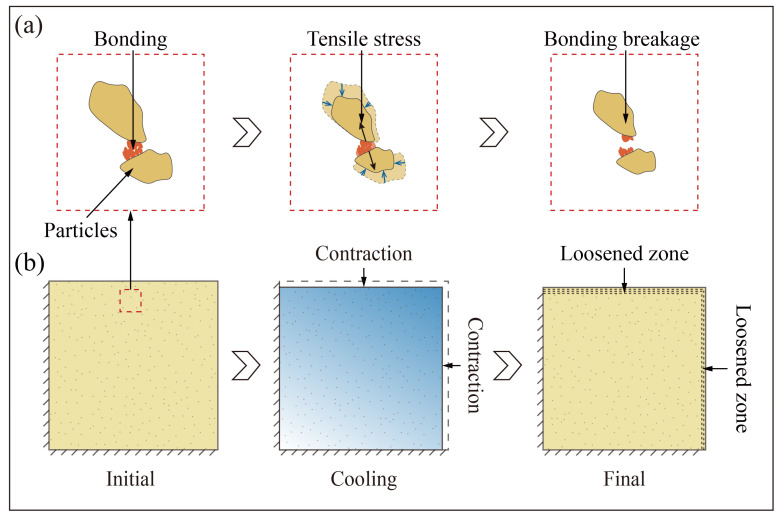
Mechanism of thermal-induced degradation caused by cold shocks at microscopic (**a**) and macroscopic (**b**) levels.

The thermal-induced tensile stresses on the soil surfaces exceed the tensile strength of the soil during the cold shock (Figure 8 and Figure 9), which could lead to structural damage to the soil surfaces. The essence of the soil damage is the loss of cohesion between soil particles [47]. The cohesion of loess is attributed to the bonding between the particles, which is facilitated by cementing agents like clay aggregation, carbonates, and soluble salts (Figure 11a) [48]. The thermal contraction between the particles generated by the sharp cooling during the thermal shock causes tensile stress. This stress instantly reaches the bonding strength, resulting in the bonding breakage (Figure 11a) [28,37,49]. This eventually leads to the formation of a loose area of topsoil (Figure 11b). The anisotropic loess structure may contribute to more loose areas on the top surface of loess than the side surface (Figure 11b). The above mechanism can lead to the formation of loose areas in the surface layer of loess slopes subjected to cold shock. These loose areas can provide a rich material basis for soil erosion activities, thus accelerating the occurrence of soil loss. Thermo-mechanical weathering as a driver of soil erosion in loess areas cannot be ignored. More attention needs to be paid to the effects of extreme temperature events on soil erosion on loess slopes.

## 5. Conclusions

Through an integrated approach combining high-resolution physical monitoring and multiphysics numerical simulations, this investigation elucidates the thermo-mechanical response mechanisms of intact loess under extreme thermal shocks. The main findings derived are as follows:

(1) Thermal transport dynamics: Soil thermal fluctuations exhibit phase-lagged synchronization with ambient variations governed by thermal convection-conduction coupling. The low thermal diffusivity of loess induces progressive thermal wave attenuation, creating a hysteresis between surface and interior temperature extrema.
(2) Gradient-stress coupling: Thermal stress evolution is fundamentally governed by the spatiotemporal distribution of thermal gradients within the soil matrix.
(3) Anisotropic stress partitioning: While the magnitudes of thermal stress remain comparable between shock types (heat and cold), their directional characteristics demonstrate discrepancy; heat shocks induce surface compression and interior tension, whereas cold shocks generate surface tension and interior compression.
(4) Cold shock-induced degradation: In contrast to thermally benign heat shocks, cold shocks generate critical surface tensile stresses exceeding the interparticle bond strength and cause loess degradation.

## Figures and Tables

**Table 1:** Properties of the tested loess sample.

Basic Physical Properties		
Density, *ρ*		1421 kg/m^3^
Water content, *w*		1.8%
Specific gravity, *G_s_*		2.70
Particle size distribution	Clay (<5 μm)	20.5%
	Silt (5–75 μm)	73.4%
	Sand (>75 μm)	6.1%
**Mechanical properties** [33]		
Elastic modulus, *E*		100 MPa
Poisson’s ratio, *μ*		0.3
**Thermal properties**		
Specific heat capacity, *c*		1216.3 J/kg·K
Thermal conductivity, *λ*		X: 0.79 W/m·k
		Y: 0.68 W/m·k
		Z: 0.93 W/m·k
Thermal expansion coefficient, *α*		X: 1.45 × 10^−5^ K^−1^
		Y: 1.51 × 10^−5^ K^−1^
		Z: 2.20 × 10^−5^ K^−1^

## Data Availability

Data are contained within the article.
